# Functional autonomy, comorbidities and cognitive decline: a mediation analysis in neurocognitive disorders

**DOI:** 10.3389/fnagi.2026.1776156

**Published:** 2026-05-13

**Authors:** Giuseppe Valente, Claudia Bauco, Carmela Bucca, Francesca Corte, Alessandra Petrillo, Daniele Tonietti, Nadia Di Sturco, Pierluigi Diotaiuti

**Affiliations:** 1 Department of Human Sciences, Society and Health, University of Cassino and Southern Lazio, Cassino, Italy; 2 Center for Cognitive Disorders and Dementia (CDCD) C/D - Unit of Geriatric, ASL Frosinone, Frosinone, Italy; 3 Center for Cognitive Disorders and Dementia (CDCD) A/B - Unit of Geriatric, ASL Frosinone, Frosinone, Italy

**Keywords:** neurocognitive disorders, cognitive decline, comorbidity, functional autonomy, instrumental activities of daily living, cognitive reserve, mediation analysis, Cumulative Illness Rating Scale

## Abstract

**Background:**

Neurocognitive Disorders (NCDs) represent a growing global health concern, significantly impacting both quality of life and healthcare systems. Emerging evidence suggests that functional autonomy, measured through Activities of Daily Living (ADL) and Instrumental Activities of Daily Living (IADL), as well as comorbidity and educational attainment, influence the trajectory of cognitive decline. However, the interplay among these factors remains insufficiently understood. This study investigates the mediating role of IADL between comorbidity and disorder severity, while examining the direct and IADL-mediated effects of educational attainment (as a proxy of cognitive reserve).

**Objectives:**

To explore the interrelationships between comorbidity (Cumulative Illness Rating Scale—CIRS), functional autonomy (ADL and IADL), educational attainment, and the severity of Neurocognitive Disorders. Specifically, the study aims to assess the mediating effect of IADL and examine how demographic and clinical variables (including age, gender, and education level) are associated with cognitive impairment.

**Methods:**

An observational study was conducted on a sample of 1,033 individuals, including patients diagnosed with major or mild Neurocognitive Disorder, as well as individuals without dementia but reporting subjective memory complaints. Functional autonomy was assessed using the ADL and IADL scales, comorbidity through the Cumulative Illness Rating Scale (CIRS), and educational attainment was measured in total years of formal schooling. Multiple linear regression analyses and mediation testing were carried out using Structural Equation Modeling (SEM).

**Results:**

Comorbidity (CIRS) was the strongest predictor of disorder severity (*B* = 0.259, *p* = 0.001), followed by age (*B* = 0.012, *p* < 0.001), educational attainment (*B* = −0.018, *p* = 0.003), and IADL scores (*B* = −0.109, *p* < 0.001). In multiple regression, diagnostic severity was significantly associated with comorbidity (CIRS; *B* = 0.259), age (*B* = 0.012), educational attainment (*B* = −0.018), and functional autonomy (IADL; *B* = −0.109). SEM supported partial mediation of the CIRS–severity association via IADL (indirect effect = 0.222), with excellent model fit (CFI = 0.95; RMSEA = 0.03).

**Conclusion:**

Comorbidity, functional autonomy, and educational attainment play critical roles in the progression of Neurocognitive Disorders. Higher comorbidity is linked to more severe cognitive deficits, whereas greater functional independence and educational background are protective factors. Incorporating ADL, IADL, and CIRS assessments into diagnostic and care planning, alongside demographic profiling, may enhance the precision of clinical interventions, support functional independence, and reduce caregiver burden.

## 1 Introduction

Neurocognitive Disorders (NCDs) are among the foremost public health challenges worldwide, with their impact steadily increasing due to population aging. The most recent estimates suggest that the number of individuals affected by these conditions will exceed 150 million by 2050, placing significant strain on both healthcare and social care systems (Nichols et al., 2022). Although Alzheimer’s disease represents the most common form of NCD, the heterogeneity in etiopathogenesis and the variability in clinical progression underscore the need for a multidimensional diagnostic and therapeutic approach (Sachdev et al., 2014; Rodriguez Then et al., 2021). In Italy, it is estimated that over 1.1 million people are affected by major Neurocognitive Disorder, with 50–60% diagnosed with Alzheimer’s disease. Approximately 900,000 individuals present with mild Neurocognitive Disorder, involving nearly 3 million people when including patients and their families (Istituto Superiore di Sanità, 2023). These figures highlight the urgent need for effective strategies aimed at early diagnosis and integrated management of these disorders.

One of the central elements in the assessment of NCDs is the distinction between isolated cognitive deficits and functional impairment. According to the DSM-5-TR, mild Neurocognitive Disorder (Minor NCD) is defined as a condition characterized by modest cognitive decline that does not significantly interfere with everyday autonomy, whereas major Neurocognitive Disorder (Major NCD) involves impairment severe enough to compromise the individual’s independence (American Psychiatric Association, 2022). However, the progression of cognitive decline can be influenced by several factors, including the degree of comorbidity, cognitive reserve, and residual functional abilities (Stern, 2002, 2012; Raimo et al., 2024; Petersen and Jack, 2020; Sperling et al., 2019).

Early identification of functional decline is crucial for both diagnosis and care planning in NCD patients. Recent studies suggest that Instrumental Activities of Daily Living (IADL) are particularly sensitive indicators of early cognitive deterioration, often preceding changes detected by traditional neuropsychological tests (Cipriani et al., 2020; Altieri et al., 2021; Libon et al., 2023).

Comorbid conditions play a critical role in the progression of cognitive decline. The Cumulative Illness Rating Scale (CIRS) is a well-established instrument used to assess the burden of chronic illnesses on patient functioning. It has shown a significant association with increased risk of cognitive impairment and loss of autonomy (Fauth and Gibbons, 2014; Temedda et al., 2024; Bidzan et al., 2023). Nevertheless, the interplay between comorbidity, cognitive function, and functional autonomy remains insufficiently explored. A more in-depth analysis of these relationships may help in developing more effective interventions, ultimately improving patients’ quality of life and reducing caregiver burden (Bunn et al., 2014; Hartle et al., 2022; Benussi et al., 2024).

In light of these considerations, the present study aims to examine whether IADL is statistically associated with the relationship between comorbidity and diagnostic severity. Specifically, our objective is to assess the mediating role of IADL in the relationship between comorbidity severity and cognitive impairment, providing novel evidence to support a more precise and personalized diagnostic framework. This integrated approach seeks to identify patients at risk of disease progression at an early stage, enabling timely and targeted interventions to slow cognitive decline and preserve functional independence.

### 1.1 Diagnostic criteria and regulatory guidelines

The diagnosis of both Minor and Major Neurocognitive Disorder (NCD) was made according to the criteria defined in the *Diagnostic and Statistical Manual of Mental Disorders, Fifth Edition, Text Revision (DSM-5-TR)*. Minor NCD is characterized by a modest decline in one or more cognitive domains (memory, attention, executive function, language, visuospatial abilities, or social cognition) that does not significantly interfere with everyday independence, although compensatory strategies may be required. In contrast, Major NCD involves a more severe cognitive decline that compromises functional autonomy and results in a substantial loss of independence (American Psychiatric Association, 2022). Differentiating between these two conditions is essential for therapeutic planning, as functional decline typically progresses, leading to increasing care needs (Sachdev et al., 2014).

In addition to the DSM-5-TR criteria, the diagnosis and clinical management of patients are guided by both national and regional healthcare protocols. The *National Dementia Plan* (Piano Nazionale delle Demenze—PND), issued by the Italian Ministry of Health in 2015, promotes an integrated care model for early diagnosis and multidisciplinary management of patients. The plan emphasizes a personalized approach aimed at slowing functional decline and supporting caregivers (Ministero della Salute, 2015).

At the regional level, the *Diagnostic-Therapeutic Care Pathway (PDTA)* for Dementia issued by the Region Lazio (2024) provides specific clinical protocols for diagnostic assessment and treatment of NCD. It emphasizes the combined evaluation of cognitive and functional impairments using standardized neuropsychological tests and neuroimaging techniques. The pathway includes test batteries for identifying distinct patterns of cognitive deterioration and integrates neuroimaging (MRI or CT scans) to help differentiate among various dementia etiologies such as Alzheimer’s disease, vascular dementia, and mixed forms (Regione Lazio, 2024).

From a therapeutic perspective, *AIFA Note 85* regulates the prescription of cholinesterase inhibitors (donepezil, rivastigmine, galantamine) and memantine, based on the severity of the disorder and the level of functional impairment. Treatment eligibility is determined by the Mini-Mental State Examination (MMSE) score, which is used to classify the severity of cognitive impairment and guide pharmacological decisions. The Note also provides for periodic monitoring to assess treatment efficacy and determine whether modifications or discontinuation are warranted based on the clinical course (AIFA, 2023).

The integration of these diagnostic and regulatory frameworks allows for a standardized pathway in the identification and management of Neurocognitive Disorders, ensuring a clinical approach that is both evidence-based and aligned with the most recent.

## 2 Materials and methods

### 2.1 Study design

This retrospective observational study included a sample of 1,033 individuals who underwent evaluation at the Cognitive Disorders and Dementia Centers (Centri per i Disturbi Cognitivi e le Demenze − CDCD) of the Local Health Authority in the province of Frosinone. All diagnostic processes were completed in the year 2024.

Inclusion criteria were: (1) age 60 years or older, (2) completion of the full diagnostic work-up at a CDCD, and (3) availability of complete clinical and functional data. Exclusion criteria included incomplete data, presence of uncontrolled major psychiatric disorders, recent acute neurological conditions, or severe sensory deficits that could interfere with cognitive and functional assessment.

Clinical and functional data were retrospectively extracted from patient medical records and anonymized in compliance with the General Data Protection Regulation (GDPR, EU Regulation 2016/679) and local ethical guidelines. The study was conducted in accordance with the Declaration of Helsinki. The requirement for approval was waived due to retrospective, fully anonymized data. Each record was assigned a unique alphanumeric identifier not linked to personal data, ensuring full de-identification of information. Diagnostic severity was treated as approximately continuous for modeling purposes. Because diagnostic severity was coded on an ordered three-level scale, we additionally conducted a sensitivity analysis using ordinal logistic regression (proportional odds model) with the same predictors included in the main linear model. Statistical analyses were conducted using JAMOVI (v2.6.16) and/or SPSS (version 16), as specified below for each analysis.

### 2.2 Instruments and measures

Standardized and widely recognized instruments were used for data collection and patient evaluation, focusing on comorbidity assessment, cognitive function, and functional autonomy:

- Cumulative Illness Rating Scale (CIRS):

The CIRS was employed to quantify the overall burden of comorbid conditions by assigning a severity score to each organ system. It is a well-established tool in geriatric practice for assessing the impact of multiple chronic conditions on overall patient functioning. The CIRS total was used as a measure of comorbidity, reflecting the average severity of the identified pathological conditions (Linn et al., 1968; Parmelee et al., 1995; Salvi et al., 2008).

- Mini-Mental State Examination (MMSE):

The MMSE was administered as a screening tool for global cognitive functioning. It evaluates several cognitive domains, including orientation, memory, attention, language, and visuospatial abilities, providing a total score useful for tracking the extent of cognitive impairment (Folstein et al., 1975; Measso et al., 1993; Magni et al., 1996).

- Activities of Daily Living (ADL):

The ADL scale measures the patient’s level of independence in performing basic self-care tasks. It assesses six essential functions: feeding, dressing, bathing, toileting, transferring, and continence. Scores range from maximum (indicating full independence) to minimum (indicating severe functional impairment) (Katz et al., 1963).

- Instrumental Activities of Daily Living (IADL):

The IADL scale evaluates more complex activities that require both cognitive and motor engagement, which are essential for independent living. The domains assessed include: telephone use, financial management, meal preparation, transportation use, medication adherence, shopping, and household management (Lawton and Brody, 1969).

In the present sample, internal consistency was estimated for the MMSE, ADL, and IADL using Cronbach’s alpha and McDonald’s omega. Reliability was acceptable for the MMSE [α = 0.75, 95% CI (0.73, 0.77); ω = 0.81, 95% CI (0.78, 0.83)], good for the ADL [α = 0.77, 95% CI (0.75, 0.79); ω = 0.87, 95% CI (0.86, 0.88)], and good for the IADL [α = 0.87, 95% CI (0.85, 0.88); ω = 0.87, 95% CI (0.86, 0.88)]. For the Cumulative Illness Rating Scale (CIRS), internal consistency coefficients such as Cronbach’s alpha and McDonald’s omega were considered less informative, as the instrument is better conceptualized as a multidimensional comorbidity index rather than a reflective psychometric scale. Therefore, the average inter-item correlation was calculated [AIC = 0.065, 95% CI (0.053, 0.076)], consistent with the heterogeneity of the medical domains assessed.

### 2.3 Sample characteristics and statistical analyses

The statistical analyses were conducted in three main phases. First, a descriptive analysis was performed to characterize the sample, including demographic, clinical, and functional variables, with particular attention to differences by gender, age, and educational level. Independent sample *t*-tests were subsequently conducted to compare diagnostic groups in terms of age and education, highlighting significant differences related to the severity of the Neurocognitive Disorder. A one-way analysis of variance (ANOVA) was used to evaluate differences in Mini-Mental State Examination (MMSE) subscale scores across diagnostic groups, revealing significant variations across cognitive domains.

Multiple linear regression analysis was then employed to assess the associations between clinical, demographic, and functional variables and the severity of Neurocognitive Disorder. Prior to conducting the regression, standard assumptions were checked to ensure model validity: residual normality was assessed via Q-Q plot and histogram, linearity was verified through residuals vs. predicted values plots, and the absence of multicollinearity was confirmed using Variance Inflation Factor (VIF ≤ 5). In addition, the Durbin-Watson test, with values close to 2, confirmed the independence of residuals (Kutner et al., 2005).

We tested a mediation model using structural equation modeling to examine whether functional autonomy (Instrumental Activities of Daily Living; IADL) mediates the associations between comorbidity burden (Cumulative Illness Rating Scale total score; CIRS) and diagnostic severity. Diagnostic severity was coded as a three-level ordered variable (0 = non-demented, 1 = Minor NCD, 2 = Major NCD) and treated as approximately continuous for modeling. Years of education was included as a predictor (proxy of cognitive reserve) with both direct effects on diagnostic severity and indirect effects via IADL. The hypothesized model specified paths from CIRS and years of education to IADL, and from IADL to diagnostic severity, while retaining direct paths from CIRS and years of education to diagnostic severity (i.e., partial mediation). CIRS and years of education were allowed to covary. Direct, indirect, and total effects were estimated, and statistical significance was evaluated using standard errors and 95% confidence intervals as reported in the corresponding effects tables. Model fit was assessed using the Comparative Fit Index (CFI) and the Root Mean Square Error of Approximation (RMSEA), with conventional thresholds indicating good fit (CFI ≥ 0.90–0.95; RMSEA ≤ 0.06–0.08) (Gunzler et al., 2013; Kline, 2023). All mediation analyses were conducted in JAMOVI (version 2.6.16) using the SEM module. Confidence intervals for indirect effects were computed using the delta method (normal-theory CI), consistent with the estimation approach implemented in the software.

## 3 Results

To describe the sample characteristics, Table 1 provides a summary of the demographic data and gender distribution. In the analyzed sample, 63.89% of participants were female (*n* = 660), while 36.11% were male (*n* = 373). The mean age of female participants was 77.59 years ( ± 8.25), slightly higher than that of males, whose mean age was 76.92 years ( ± 8.10). The total sample consisted of 1,033 individuals, with a combined mean age of 77.28 years ( ± 8.21).

**TABLE 1:** Gender distribution and mean age of the sample.

Gender	*n*	%	Mean age (SD)
Female	660	63.89	77.59 (8.25)
Male	373	36.11	76.92 (8.10)
Total	1,033	100.00	77.28 (8.21)

The distribution of education levels in the analyzed sample highlights a predominance of lower educational levels, with marked gender differences (see Table 2). Among women, 43.33% (*n* = 286) had completed only primary education, compared to 27.61% (*n* = 103) of men. The “no formal education” category included 18.33% (*n* = 121) of female participants and 5.01% (*n* = 19) of males. Conversely, men were more represented at higher levels of education: 20.64% (*n* = 77) of men had completed upper secondary school, compared to 15.30% (*n* = 101) of women; 6.70% (*n* = 25) of men held a university degree or equivalent, compared to 3.03% (*n* = 20) of women. Primary education was the most common qualification, accounting for 37.66% (*n* = 389) of the total sample. These findings suggest a gender disparity in educational attainment, with a higher concentration of men in the upper tiers of the educational spectrum.

**TABLE 2:** Educational level distribution by gender.

Gender	No formal education	Primary school	Lower secondary school	Upper secondary school	Professional diploma	University degree
Female	121(18.33%)	286(43.33%)	113(17.12%)	101(15.30%)	19(2.88%)	20(3.03%)
Male	19(5.01%)	103(27.61%)	131(35.12%)	77(20.64%)	17(4.56%)	25(6.70%)
Total	140(13.55%)	389(37.66%)	244(23.52%)	177(17.14%)	36(3.49%)	45(4.36%)

The diagnostic distribution within the sample reveals that the majority of participants (57.2%) were classified as having Major Neurocognitive Disorder (Major NCD), followed by 22.74% with Minor NCD, and 20.06% categorized as “non-demented.”

When examining demographic characteristics by diagnosis, the largest group, participants with Major NCD, had a mean age of 79.96 years (SD = 7.05), indicating a higher prevalence of this condition among older individuals. Participants without a diagnosis of Neurocognitive Disorder were younger, with a mean age of 72.09 years (SD = 8.63), while those with Minor NCD fell in between, with a mean age of 74.63 years (SD = 7.60). These results suggest a progressive association between advancing age and increasing severity of cognitive decline.

To assess the statistical significance of associations between disorder severity and demographic features, two independent sample *t*-tests were conducted. The analysis showed that participants with Major NCD were significantly older than those without a diagnosis of Neurocognitive Disorder (*t* = 11.67, *p* < 0.001). Individuals with Major NCD had significantly lower educational levels, with a mean of 6.14 years of formal education (SD = 4.38), compared to 8.96 years (SD = 4.26) in the non-demented group (*t* = −8.03, *p* < 0.001).

**FIGURE 1:**
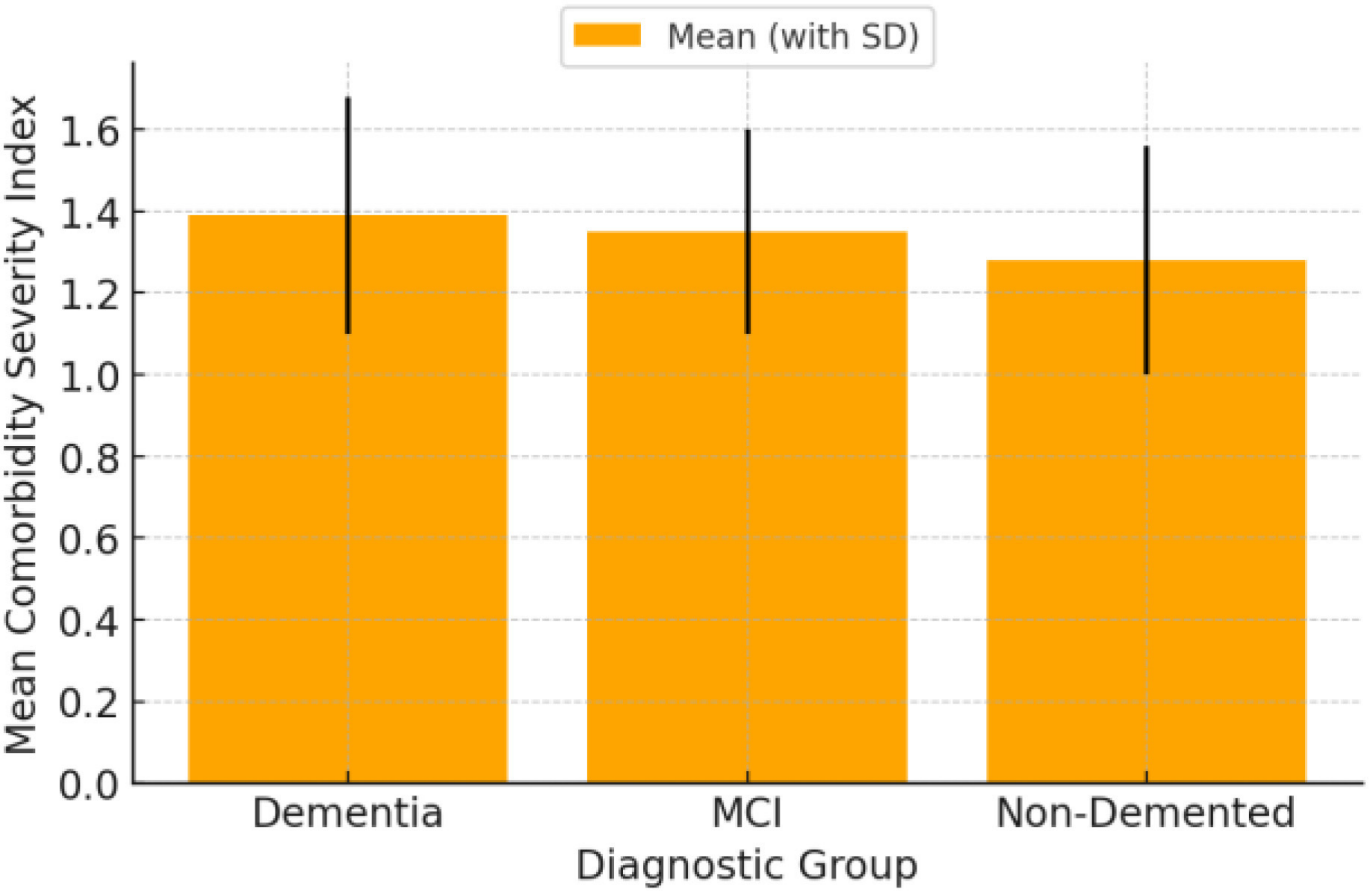
Mean comorbidity index by diagnostic group. Flow chart of participant selection and analytic sample derivation. The diagram summarizes the initial pool of consecutive patients evaluated at the Center, the inclusion/exclusion steps, and the final sample included in descriptive analyses, regression, and SEM (*N* = 1,033).

Further descriptive analyses were performed to explore the severity of the disorder using three key indicators: the CIRS total (comorbidity burden), the total score on the Mini-Mental State Examination (MMSE), and functional autonomy levels as assessed by the ADL and IADL scales. The analysis revealed significant differences in the mean comorbidity index across diagnostic groups. Patients with Major NCD exhibited the highest mean CIRS total (1.39, SD = 0.29), compared to those with Minor NCD (1.35, SD = 0.25; *p* = 0.035) and those without a Neurocognitive Disorder (1.28, SD = 0.28; *p* < 0.001). Figure 1 clearly illustrates these differences, with the Major NCD group showing consistently higher average values.

**FIGURE 2:**
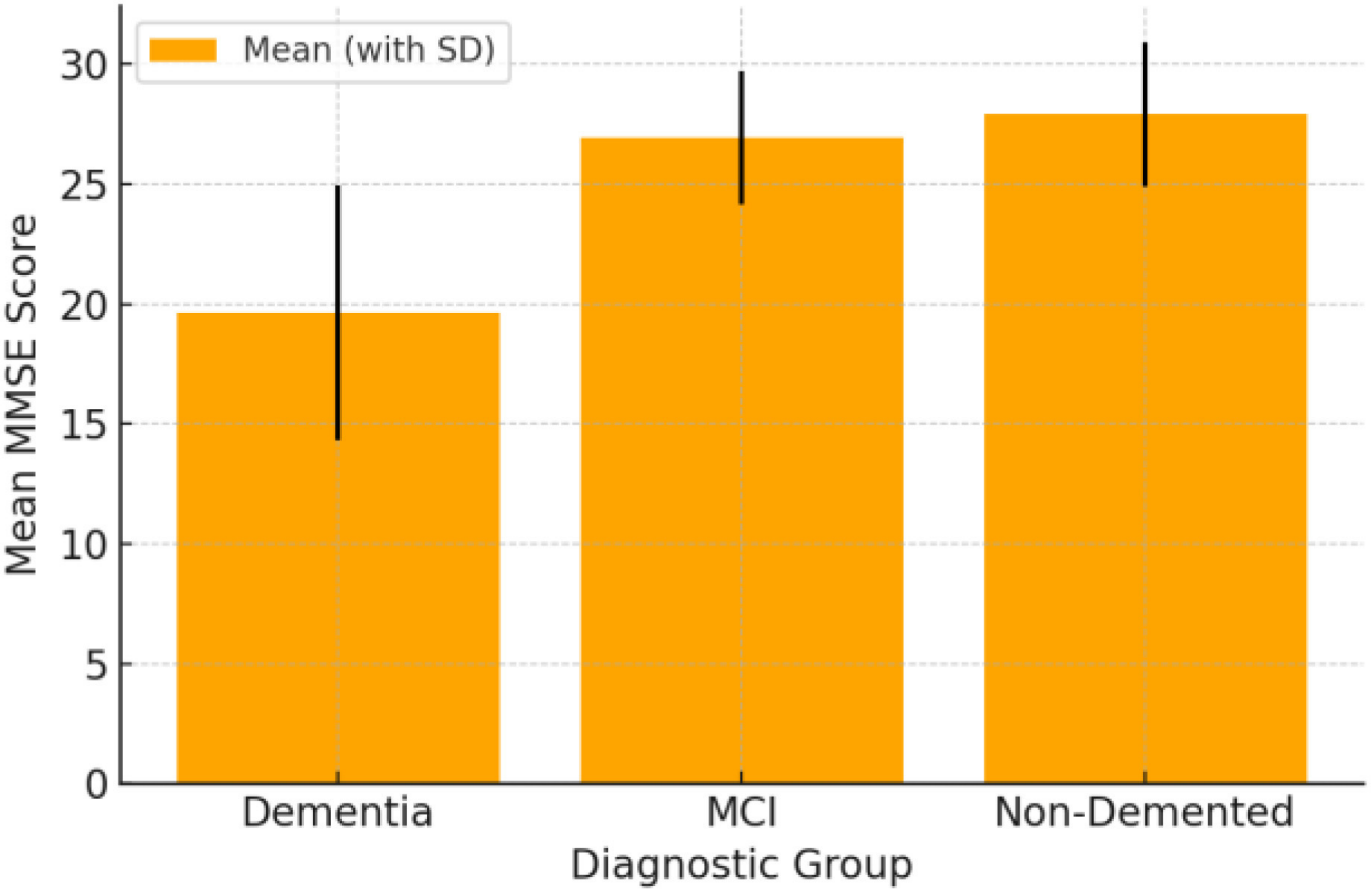
Mean MMSE score by diagnostic group. Group differences in cognitive performance (MMSE) across diagnostic severity levels (non-demented, Minor NCD, Major NCD). Bars represent mean values with dispersion (SD). Between-group comparisons were tested using one-way ANOVA with appropriate *post-hoc* pairwise tests (reported in text); higher diagnostic severity was associated with lower MMSE scores.

Regarding MMSE scores, patients with Major Neurocognitive Disorder reported significantly lower mean scores (19.61, SD = 5.31) compared to those with Minor NCD (26.94, SD = 2.78; *p* < 0.001) and individuals without a diagnosis of Neurocognitive Disorder (27.90, SD = 3.00; *p* < 0.001). Figure 2 provides a visual representation of these differences, clearly illustrating the progressive decline in MMSE scores corresponding to increasing severity of cognitive impairment.

**FIGURE 3:**
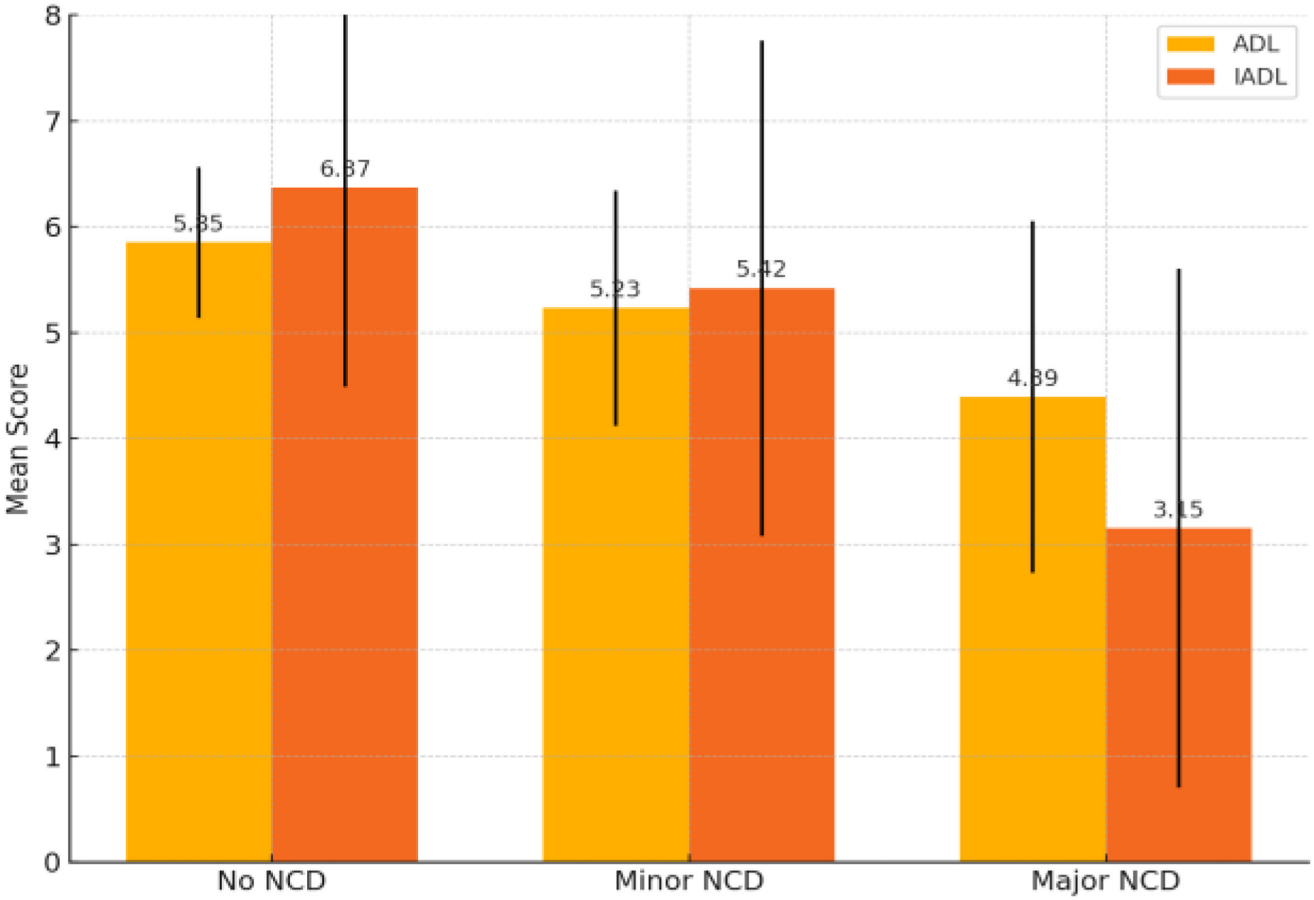
Mean ADL and IADL scores by diagnostic group. Group differences in functional autonomy (IADL total score) across diagnostic severity levels (non-demented, Minor NCD, Major NCD). Bars represent mean values with dispersion (SD). Between-group comparisons were tested using one-way ANOVA with *post-hoc* pairwise tests (reported in text); greater diagnostic severity was associated with poorer functional autonomy (lower IADL scores).

Autonomy, assessed through the ADL and IADL scales, also showed a decline in scores among patients with Major Neurocognitive Disorder compared to those with Minor NCD and individuals without a Neurocognitive Disorder diagnosis. For ADL, patients with Major NCD had a mean score of 4.39 (SD = 1.66), while for IADL the mean score was 3.15 (SD = 2.45). Figure 3 illustrates the differences in mean ADL and IADL scores, showing a progressive decline in functional autonomy as cognitive impairment severity increases.

To evaluate the relative contribution of clinical, demographic, and functional variables to the severity of Neurocognitive Disorder, a multiple linear regression analysis was performed. Prior to conducting the regression, key assumptions were verified to ensure the validity of the results. Residual analysis revealed an approximately normal distribution, as confirmed by both the Q-Q plot and the histogram of residuals. The relationship between the independent variables and the dependent variable was found to be linear, as shown by the residuals-versus-fitted-values plot, which did not display any systematic patterns. The Durbin-Watson statistic (1.61) suggested a near absence of autocorrelation among residuals, confirming their independence. Additionally, analysis of the Variance Inflation Factor (VIF) ruled out multicollinearity, with all VIF values below 1.5.

As shown in Table 3, the comorbidity index (Cumulative Illness Rating Scale, CIRS) emerged as the most significant predictor of diagnostic severity (*B* = 0.259, *p* = 0.001). Other significant predictors included the total score on the Instrumental Activities of Daily Living scale (IADL; *B* = −0.109, *p* < 0.001), years of formal education (*B* = −0.018, *p* = 0.003), and age (*B* = 0.012, *p* < 0.001).

**TABLE 3:** Multiple linear regression predicting diagnostic severity.

Predictor	B (Unstandardized)	SE	β (Standardized)	t	*p*
Intercept	1.753	0.292	—	5.999	< 0.001
Age	0.012	0.003	0.126	3.814	< .001
Years of Education	−0.018	0.006	−0.087	−2.940	0.003
CIRS total	0.259	0.079	0.092	3.271	0.001
IADL total	−0.109	0.009	−0.371	−11.895	< .001

Model fit. *R*^2^ = 0.247; Durbin–Watson = 1.61; all VIF < 1.5.

The regression model accounted for 24.7% of the variance in the severity of Neurocognitive Disorder (*R*^2^ = 0.247), indicating that while the variables considered had a significant effect, other unmeasured factors may also contribute. Specifically, higher functional autonomy (as measured by IADL scores) and lower comorbidity (CIRS) were associated with reduced disorder severity, whereas older age and lower educational attainment were linked to increased cognitive impairment.

**FIGURE 4:**
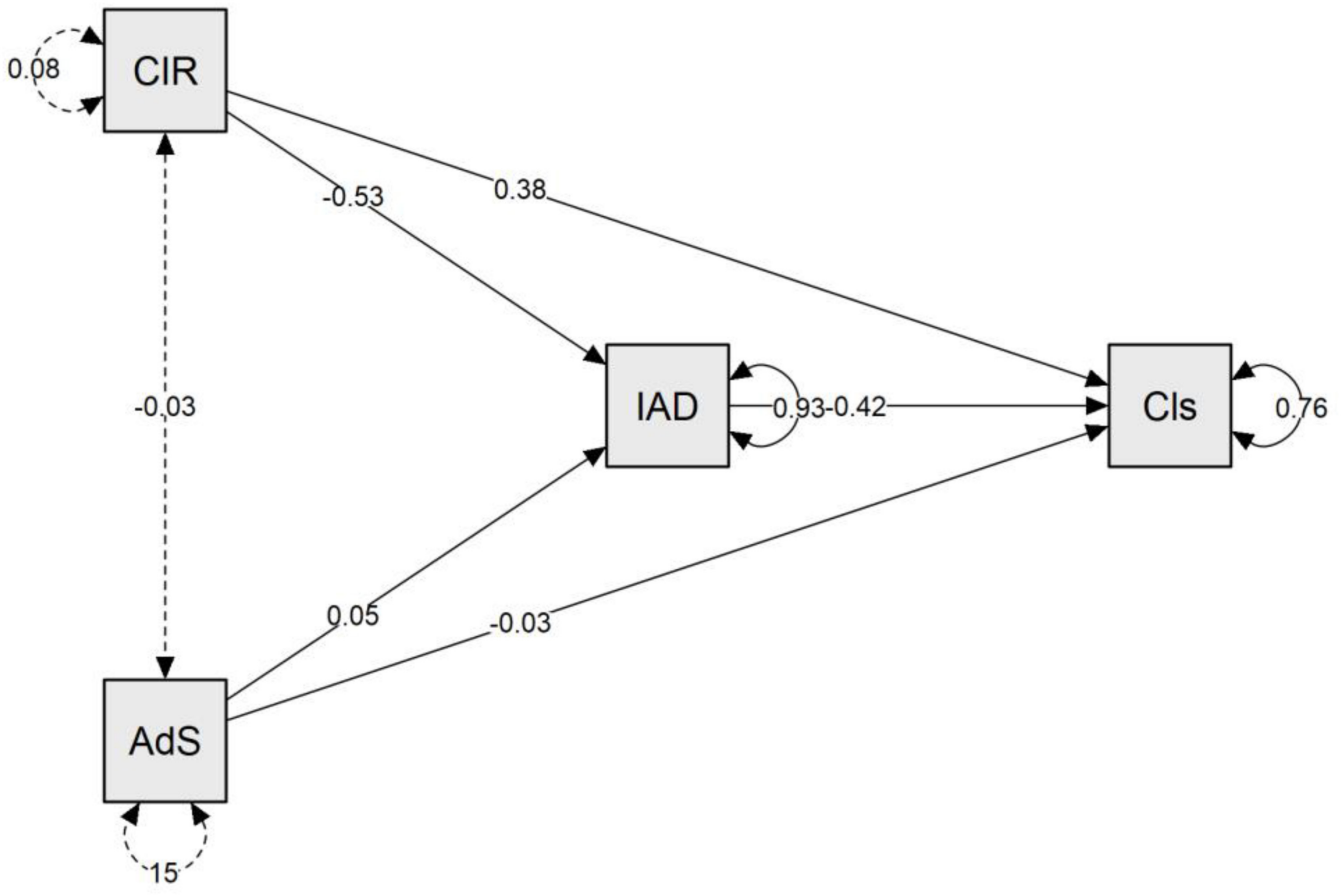
Mediation model.

Structural equation modeling (SEM) was used to test a mediation model in which functional autonomy (IADL) mediates the relationships between comorbidity (CIRS), educational attainment (years of schooling), and diagnostic severity (Figure 4). As shown in the structural path estimates (Table 4), higher comorbidity was associated with poorer functional autonomy (CIRS → IADL: *B* = −0.53), and lower functional autonomy predicted greater diagnostic severity (IADL → severity: *B* = −0.42). Comorbidity also showed a significant direct effect on diagnostic severity (*B* = 0.377, SE = 0.098, *z* = 3.863, *p* < 0.001; Table 4a), indicating that the association was partially mediated by IADL. The indirect effect of CIRS on diagnostic severity via IADL was significant (indirect effect = 0.222, SE = 0.047, *z* = 4.687, *p* < 0.001; Table 4b), yielding a significant total effect (total effect = 0.599, SE = 0.106, *z* = 5.631, *p* < 0.001; Table 4c).

**TABLE 4:** SEM structural path estimates (mediation model).

Structural path	Estimate (B)	Interpretation (direction)
CIRS total → IADL total (*a1*)	−0.53	Higher comorbidity → lower functional autonomy
Years of Education → IADL total (*a2*)	0.05	Higher education → better functional autonomy
IADL total → Diagnostic severity (*b*)	−0.42	Better autonomy → lower diagnostic severity
CIRS total → Diagnostic severity (*c’1*)	0.38	Direct adverse effect of comorbidity on severity
Years of Education → Diagnostic severity (*c’2*)	−0.03	Direct protective effect of education on severity
CIRS total ↔ Years of Education (covariance)	−0.03	Small negative association between predictors

Unstandardized coefficients are reported; inferential statistics for direct/indirect/total effects are provided in Tables 4a–c.

**TABLE 4a:** Direct effects (SEM; ML).

Predictor → outcome	Estimate	Std. error	*z*	*p*	95% CI (lower)	95% CI (upper)
CIRS total → diagnostic severity	0.377	0.098	3.863	< 0.001	0.186	0.568
Years of education → diagnostic severity	−0.031	0.007	−4.281	< 0.001	−0.045	−0.017

Delta method standard errors, normal theory confidence intervals, ML estimator.

**TABLE 4b:** Indirect effects (SEM; ML).

Indirect path	Estimate	Std. error	*z*	*p*	95% CI (lower)	95% CI (upper)
CIRS total → IADL total → diagnostic severity	0.222	0.047	4.687	< .001	0.129	0.315
Years of education → IADL total → diagnostic severity	−0.022	0.004	−6.067	< .001	−0.029	−0.015

Delta method standard errors, normal theory confidence intervals, ML estimator.

**TABLE 4c:** Total effects (SEM; ML).

Predictor → outcome	Estimate	Std. error	z	p	95% CI (lower)	95% CI (upper)
CIRS total → diagnostic severity	0.599	0.106	5.631	< 0.001	0.390	0.807
Years of education → diagnostic severity	−0.052	0.008	−6.776	< 0.001	−0.068	−0.037

Delta method standard errors, normal theory confidence intervals, ML estimator.

Educational attainment showed a protective association with diagnostic severity both directly (*B* = −0.031, SE = 0.007, *z* = −4.281, *p* < 0.001; Table 4a) and indirectly via IADL (indirect effect = −0.022, SE = 0.004, z = −6.067, *p* < 0.001; Table 4b), with a significant total effect (total effect = −0.052, SE = 0.008, *z* = −6.776, *p* < 0.001; Table 4c). Overall model fit indicated an excellent fit to the data (CFI = 0.95; RMSEA = 0.03).

Because diagnostic severity is inherently ordinal, we additionally fitted an ordinal logistic regression model as a sensitivity analysis. This analysis confirmed the main findings, showing that older age (*b* = 0.045, *p* < 0.001), higher CIRS total (*b* = 0.917, *p* = 0.002), lower educational attainment (*b* = −0.079, *p* < .001), and lower IADL total (*b* = −0.389, *p* < 0.001) were significantly associated with greater diagnostic severity. However, because diagnostic severity in Neurocognitive Disorders is partly defined by loss of everyday functional independence, the association involving IADL, particularly its indirect role in the mediation model, should be interpreted cautiously, as some degree of conceptual overlap between functional autonomy and the outcome is likely.

## 4 Discussion

The findings of this study provide an in-depth overview of the variables that influence the severity of Neurocognitive Disorders, highlighting the central role of comorbidity, functional autonomy, and educational attainment. Data analysis confirmed that higher levels of comorbidity are associated with greater cognitive and functional impairment, suggesting that chronic medical conditions may accelerate cognitive decline (Koyanagi et al., 2018; Valdevila Figueira et al., 2024).

At the same time, the preservation of functional autonomy, particularly in Instrumental Activities of Daily Living (IADL), emerged as a key factor in mitigating the negative effects of comorbidities and in maintaining patients’ independence (Nourbakhsh et al., 2017; Altieri et al., 2021). Educational level also demonstrated a protective effect, consistent with the concept of cognitive reserve, whereby higher lifelong cognitive stimulation may delay the onset and progression of cognitive deterioration (Godinho et al., 2022).

Descriptive analysis revealed significant gender differences, with women showing a higher prevalence of lower educational levels compared to men. This finding aligns with previous studies that have highlighted the impact of education on cognitive reserve and the ability to cope with neurocognitive decline (Wilson et al., 2019). Comparisons across diagnostic groups confirmed that patients with Major Neurocognitive Disorder scored significantly lower on cognitive tests, particularly in episodic memory and attention tasks (MMSE), supporting neuropsychological models that identify these domains as key markers of the disorder (Li et al., 2016; Tahami Monfared et al., 2022).

The analysis of functional autonomy reinforced the evidence that declines in IADL typically precede those in ADL, suggesting that IADL may serve as sensitive indicators of early cognitive impairment (Passler et al., 2020).

One of the most clinically relevant aspects emerging from this study relates to the practical application of these findings in light of the most recent national and regional guidelines. The 2024 Dementia Diagnostic-Therapeutic Care Pathway (PDTA) from the Lazio Region emphasizes the importance of a combined assessment of cognitive and functional deficits for more accurate diagnosis and more effective management of patients with Neurocognitive Disorders. In this context, our study provides empirical support for integrating functional assessment tools, such as ADL and IADL, into the diagnostic process. Although diagnostic severity is clinically defined partly by functional impairment, our findings highlight the practical value of systematically quantifying IADL as an early marker and as a target for interventions.

Systematic inclusion of functional autonomy assessments, as recommended by the PDTA, may allow for earlier and more targeted patient management, optimizing therapeutic interventions aimed at preserving independence for as long as possible and reducing caregiver burden, both physical and emotional (Dauphinot et al., 2016; Temedda et al., 2024).

The regression analysis confirmed the key role of clinical and functional variables in determining the severity of Neurocognitive Disorders. Comorbidity level, older age, lower educational attainment, and impaired functional autonomy emerged as significant predictors of cognitive impairment. Notably, the mediation model revealed that IADL showed a significant statistical indirect association in the relationship between comorbidity and diagnostic severity, suggesting that intervention strategies aimed at preserving functional autonomy may be clinically relevant, although the present cross-sectional data do not allow conclusions about their effect on the course of cognitive decline. This finding holds important clinical implications and aligns with recent studies (Nicolas et al., 2021; Jang et al., 2022), indicating that rehabilitation and support programs focused on maintaining IADL could have a significant impact on the progression of Neurocognitive Disorders.

Although the study is supported by a solid methodological framework, certain limitations must be acknowledged. The cross-sectional nature of the analysis does not allow for definitive causal inferences among the variables. Future longitudinal studies will be essential to better understand the temporal dynamics of disorder progression and to evaluate the effectiveness of specific interventions. Severity classification is partly based on functional criteria; therefore, mediation effects involving IADL may be overestimated. Because diagnostic severity partly reflects functional impairment, mediation effects involving IADL may be inflated; nonetheless, the findings support routine IADL assessment as a clinically actionable marker for risk stratification and care planning.

While the proposed model explains a significant proportion of the variance in Neurocognitive Disorder severity, it does not account for some variables previously identified in the literature as potential predictors of cognitive decline. In particular, lifestyle factors such as smoking and physical activity were not included in this study, despite prior associations with cognitive deterioration (Sousa et al., 2020). Although key clinical and functional variables were included, other factors, such as social support, lifestyle behaviors, and mood, may influence the trajectory of cognitive decline and warrant further investigation (Adriani et al., 2024).

The integration of neurodegeneration biomarkers with clinical and functional assessments represents a promising approach to enhance patient stratification and identify subgroups at higher risk of disease progression (Frisoni et al., 2017).

## 5 Limitations

An important conceptual limitation of the present mediation model should be acknowledged more explicitly. Functional autonomy, as measured by IADL, is closely related to the clinical definition of diagnostic severity in Neurocognitive Disorders. In particular, the distinction between Minor and Major NCD partly depends on the extent to which cognitive impairment interferes with independent daily functioning. As a result, the inclusion of IADL as a mediator of diagnostic severity may introduce conceptual overlap between the mediator and the outcome, potentially inflating the magnitude of the indirect association observed in the SEM analyses. Therefore, the mediation findings should not be interpreted as evidence of a distinct causal pathway, but rather as an indication of a statistical pattern of association among comorbidity, functional autonomy, and diagnostic severity in this clinical sample.

A second major limitation concerns the cross-sectional design. Because all variables were measured at the same time point, no temporal ordering can be established, and causal interpretations are not warranted. Accordingly, the observed associations should be interpreted as concurrent relationships rather than as evidence that comorbidity leads to reduced functional autonomy or that preserving IADL slows the progression of cognitive impairment. Longitudinal studies will be necessary to clarify the temporal dynamics among comorbidity burden, functional decline, and worsening diagnostic status.

Third, diagnostic severity was modeled as a continuous variable despite its ordinal nature. However, sensitivity analyses using ordinal models yielded consistent results, supporting the robustness of the findings.

Fourth, although the sample size was large, the study was based on retrospective clinical data collected in a single healthcare setting, which may limit the generalizability of the findings. Finally, some potentially relevant variables, such as lifestyle factors, mood symptoms, and biomarkers of neurodegeneration, were not included in the model and should be considered in future studies.

Despite these limitations, the findings remain clinically informative, as they support the relevance of systematically assessing comorbidity and functional autonomy in patients with neurocognitive complaints. However, the present results should be understood primarily as supporting risk stratification and clinical profiling, rather than causal inferences about disease mechanisms or intervention effects.

## 6 Conclusion

This study highlights the importance of functional autonomy in mediating the relationship between comorbidity and the severity of Neurocognitive Disorder, reinforcing the idea that a multidimensional approach can enhance the clinical understanding and management of these conditions. The findings support the integration of functional assessment into diagnostic pathways, as recommended by the 2024 Dementia Diagnostic-Therapeutic Care Pathway (PDTA) of the Lazio Region, and emphasize the need for targeted interventions aimed at preserving independence in daily activities.

Adopting a care model that incorporates the joint assessment of cognitive, functional, and clinical factors may allow for earlier identification of patients at risk of disease progression. Specifically, our study suggests that monitoring IADL performance may be useful for identifying patients with greater clinical vulnerability and for informing timely supportive care planning of rehabilitative and support interventions (Marshall et al., 2012; Yemm et al., 2021).

Future research should further explore the role of functional autonomy through longitudinal studies and evaluate the effectiveness of specific interventions to preserve independence and prevent disease progression. Combining clinical and functional assessments with biomarkers may offer an innovative strategy to improve diagnostic accuracy and optimize treatment pathways.

In summary, our findings underscore the need for an integrated approach to the assessment and management of neurocognitive disorders, in which functional autonomy plays a central role. The implementation of intervention strategies focused on maintaining independence is clinically plausible and worthy of further investigation, but its effectiveness cannot be inferred from the present cross-sectional findings. The mediation model should therefore be interpreted as descriptive of covariance structure rather than as evidence of a mechanistic or causal process.

## Data Availability

The raw data supporting the conclusions of this article will be made available by the authors, without undue reservation.
